# Macrophage elastase derived from adventitial macrophages modulates aortic remodeling

**DOI:** 10.3389/fcell.2022.1097137

**Published:** 2023-01-10

**Authors:** Yajie Chen, Xiawen Yang, Shuji Kitajima, Longquan Quan, Yao Wang, Maobi Zhu, Enqi Liu, Liangxue Lai, Haizhao Yan, Jianglin Fan

**Affiliations:** ^1^ Guangdong Province Key Laboratory, Southern China Institute of Large Animal Models for Biomedicine, School of Biotechnology and Health Sciences, Wuyi University, Jiangmen, China; ^2^ Department of Molecular Pathology, Interdisciplinary Graduate School of Medicine, University of Yamanashi, Yamanashi, Japan; ^3^ Analytical Research Center for Experimental Sciences, Saga University, Saga, Japan; ^4^ College of Animal Science and Technology, China Agricultural University, Beijing, China; ^5^ Research Institute of Atherosclerotic Disease and Laboratory Animal Center, Xi’an Jiaotong University School of Medicine, Xi’an, China; ^6^ Key Laboratory of Regenerative Biology, South China Institute for Stem Cell, Biology and Regenerative Medicine, Guangzhou Institutes of Biomedicine and Health, Chinese Academy of Sciences, Guangzhou, China

**Keywords:** MMP-12, macrophage, elastin, abdominal aortic aneurysm, transgenic rabbits, atherosclerosis

## Abstract

Abdominal aortic aneurysm (AAA) is pathologically characterized by intimal atherosclerosis, disruption and attenuation of the elastic media, and adventitial inflammatory infiltrates. Although all these pathological events are possibly involved in the pathogenesis of AAA, the functional roles contributed by adventitial inflammatory macrophages have not been fully documented. Recent studies have revealed that increased expression of matrix metalloproteinase-12 (MMP-12) derived from macrophages may be particularly important in the pathogenesis of both atherosclerosis and AAA. In the current study, we developed a carrageenan-induced abdominal aortic adventitial inflammatory model in hypercholesterolemic rabbits and evaluated the effect of adventitial macrophage accumulation on the aortic remodeling with special reference to the influence of increased expression of MMP-12. To accomplish this, we compared the carrageenan-induced aortic lesions of transgenic (Tg) rabbits that expressed high levels of MMP-12 in the macrophage lineage to those of non-Tg rabbits. We found that the aortic medial and adventitial lesions of Tg rabbits were greater in degree than those of non-Tg rabbits, with the increased infiltration of macrophages and prominent destruction of elastic lamellae accompanied by the frequent appearance of dilated lesions, while the intimal lesions were slightly increased. Enhanced aortic lesions in Tg rabbits were focally associated with increased dilation of the aortic lumens. RT-PCR and Western blotting revealed high levels of MMP-12 in the lesions of Tg rabbits that were accompanied by elevated levels of MMP-2 and -3, which was caused by increased number of macrophages. Our results suggest that adventitial inflammation constitutes a major stimulus to aortic remodeling and increased expression of MMP-12 secreted from adventitial macrophages plays an important role in the pathogenesis of vascular diseases such as AAA.

## 1 Introduction

Abdominal aortic aneurysm (AAA) is thought to be a degenerative process affecting the aortic wall; however, its cause remains unclear (Thompson et al., 2006; Wassef et al., 2007). Regardless of etiology, the basic pathologic features of most AAA are quite similar: intimal atherosclerosis and marked destruction of the aortic wall characterized by the degradation of elastin and collagen in medial and adventitial lesions. Accumulating evidence has suggested that increased activity of matrix metalloproteinases (MMPs) derived from vascular cells such as macrophages and smooth muscle cells plays a major role in the pathogenesis of AAA (Thompson and Parks, 1996; Shah, 1997; Liapis and Paraskevas, 2003). So far, many MMPs have been detected in the lesions of AAA and it seems that many of them, either predominantly or collaboratively, are involved in the process of aneurysm’s formation. In the aortic wall, elastin and collagen constitute most of the extracellular matrix (ECM), and therefore increased elastolytic activity has been suggested to be critical in the cascade of MMP-mediated degradation of ECM. Curci and coworkers first reported that the levels of macrophage elastase (MMP-12), the major elastin-degrading enzyme in the arterial wall is remarkably increased in the lesions of AAA patients, suggesting MMP-12 to be an important MMP that is involved in the pathogenesis of AAA (Curci et al., 1998). In MMP-12 knock-out mice, a deficiency of MMP-12 attenuated calcium chloride-induced AAA (Longo et al., 2005).

MMP-12, also called macrophage metalloelastase, was first identified as a potent elastolytic metalloproteinase specifically secreted by macrophages (Banda and Werb, 1981; Shapiro et al., 1993). In addition to elastin, MMP-12 is also able to degrade a broad spectrum of components of the ECM in the arterial wall such as collagen type IV, fibronectin, laminin, vitronectin, proteoglycans and plasminogen (Chandler et al., 1996; Gronski et al., 1997). Therefore, it is very likely that in the arterial wall, MMP-12 degrade not only elastin but also collagen directly or through interaction with other MMPs. Several lines of evidence show that MMP-12 can activate other MMPs such as MMP-2 and MMP-3 (Matsumoto et al., 1998) thus, MMP-12 has a pivotal role in the activation of other MMPs in the arterial wall.

To elucidate the functional roles of MMP-12, our laboratory generated transgenic rabbits with high levels of human MMP-12 specifically in tissue macrophages (Fan et al., 2004). Using this unique model, we have demonstrated that increased MMP-12 expression enhances the development of atherosclerosis (Liang et al., 2006) and inflammatory arthritis (Wang et al., 2004). We postulated that abnormal expression of macrophage-derived MMP-12, in concert with other MMPs, may also play a central role in the abdominal aortic remodeling. Rabbits are an appropriate model to study hyperlipidemia and atherosclerosis because they are sensitive to a cholesterol diet and rapidly develop atherosclerosis (Fan et al., 2015). In addition, our previous study revealed that many MMPs are upregulated in the lesions of aortic atherosclerosis of hypercholesterolemic rabbits (Fan et al., 2018). However, cholesterol-diet manipulation alone in rabbits usually induces prominent atherosclerotic lesions initiated in the aortic arch and thoracic aorta rather than abdominal aorta, which hampers examination of the effect of MMP-12 on AAA and aortic remodeling. In the current study, we produced a model of abdominal aortic injury in rabbits fed a cholesterol diet. Through embedding carrageenan, chronic adventitial inflammation of the abdominal aorta was induced to examine whether the adventitial inflammation is involved in aortic remodeling. Here we report that MMP-12 derived from adventitial inflammatory macrophages significantly modulates the formation of aortic lesions and aortic remodeling.

## 2 Materials and methods

### 2.1 Animals

**FIGURE 1:**
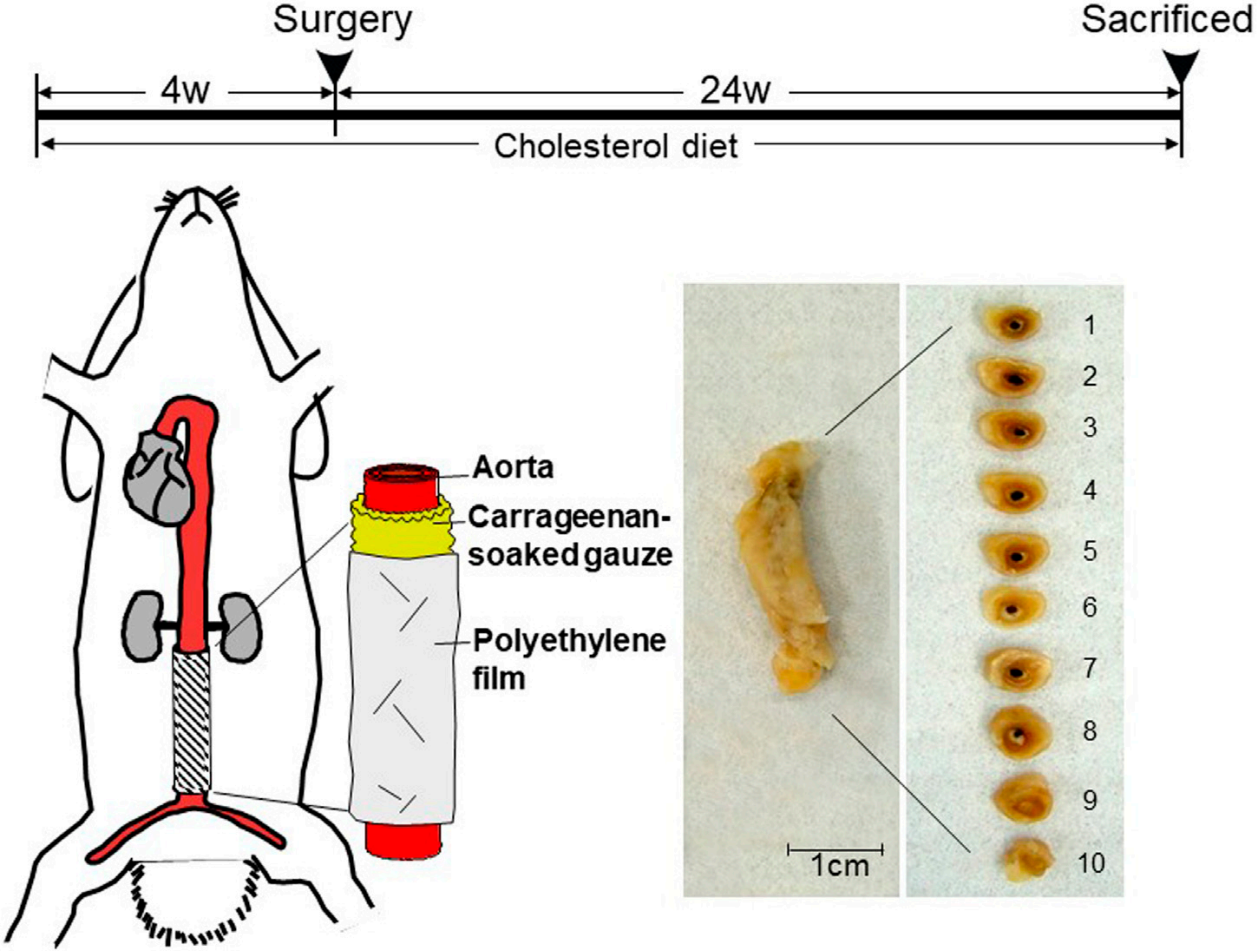
Schematic illustration of carrageenan-induced abdominal aortic lesions. Rabbits were fed a cholesterol-diet for 4 weeks before the surgery. A piece of sterilized gauze pre-soaked with λ-carrageenan was placed along the isolated abdominal aorta between the inferior mesenteric artery and lumbar artery and wrapped with a piece of polyethylene film. At the end of experiments, aortic rings were sectioned as shown on the right. The abdominal aorta was cut into 10 cross-sections.

MMP-12 transgenic (Tg) rabbits expressing human MMP-12 were generated in our laboratory as described previously (Fan et al., 2004). The Tg construct consisted of human MMP-12 cDNA (catalytic domain sequence) under the control of the human scavenger receptor A enhancer/promoter, which directs the expression of the transgene in the macrophage lineage (Fan et al., 2004) and foam cells of atherosclerotic lesions (Horvai et al., 1995). A total of 18 male Tg and 18 non-Tg littermates (5–6 months old) were used for the current study. They were fed a diet containing .3–.8% cholesterol and 3% soybean oil for 4 weeks and then, the abdominal aortic adventitial lesions were produced as described below (Figure 1). During the period of feeding, we maintained the plasma total cholesterol levels in these rabbits at a constant 800–1,000 mg/dL (Supplemental Fig.1) (similar to levels in homozygous familial hypercholesterolemic patients) by adjusting the cholesterol content of the diet.

### 2.2 Carrageenan-induced abdominal aortic adventitial lesions

To produce the abdominal aortic adventitial inflammation, we embedded carrageenan (a polysaccharide) around the isolated abdominal aorta segment (2.5 cm between inferior mesenteric artery and lumbar artery) as shown in Figure 1. Carrageenan is a potent chemoattractant for monocytes and can induce the focal accumulation of inflammatory macrophages (Wang et al., 2004). For this undertaking, rabbits were anesthetized by intramuscular injection of ketamine (25 mg/kg BW) + medetomidine hydrochloride (.5 mg/kg BW) and the abdominal aortas were exposed. A piece of sterilized gauze was pre-soaked in 10 mg/mL of λ-carrageenan (Wako Chemicals, Osaka, Japan) in .9% NaCl (w/v) at 40°C overnight, laid around the isolated abdominal aorta without any arterial branches, and gently covered with a sheet of polyethylene film to prevent inflammatory adhesion to the surrounding tissue. All rabbits were sacrificed at 24 weeks after surgery by intravenous injection of an overdose of sodium pentobarbital solution. The whole abdominal aortas were carefully collected for histological examination, immunostaining, and RNA and protein extraction (see below). This study was approved by the Animal Care Committee of the University of Yamanashi and Saga University and conformed to the Guide for the Care and Use of Laboratory Animals published by the NIH.

### 2.3 Histological examinations and immunohistochemical staining

The abdominal aortas were divided into 10 segments, fixed in 10% neutral buffered formalin, and embedded in paraffin. The sections (3 μm thick) were stained with hematoxylin and eosin (H&E) and Elastica van Gieson (EVG) for histological examination and morphometric analysis. To assess the degree to which elastic fibers were degraded, we quantified the contents of elastic fibers (expressed as elastic staining area μm (Wassef et al., 2007) per section) and the aortic diameter (expressed as μm calculated by area of the internal elastic lamina) using EVG-stained specimens and an image analysis system (Liang et al., 2006). The number of focal micro-dilated lesions in each aorta was calculated by two independent researchers. Immunohistochemical staining was performed using labeled streptavidin biotin kits (Nichirei Co, Tokyo, Japan) according to the manufacturer’s instructions. After hydration and the blocking of endogenous peroxidase activity, the sections were incubated with monoclonal antibodies (mAbs) against rabbit macrophages (RAM11, 1:200) from Dako Corporation (Carpineteria, CA), and against smooth muscle α-actin (HHF35, 1:400) and the human MMP-12 catalytic domain (MAB 919, 1:20) from R&D System (Minneapolis, MN). Non-specific mouse IgG was used to stain the sections as a negative control. Macrophage infiltration in the peri-adventitial area was quantitated using an image analysis system after immunohistochemical staining. The positive staining area of the whole adventitial granulomatous lesion was measured and expressed as the percentages of the lesions. In addition, *in situ* β-casein zymography was performed using selected frozen sections to confirm MMP enzymatic activity in adventitial macrophages (Yu et al., 2008).

### 2.4 Real-time reverse-transcription polymerase chain reaction

A 1.0 μg sample of total RNA isolated from the aortas of three non-Tg and three Tg rabbits was transcribed into cDNA using QuantiTect^®^ reverse transcription kit (Qiagen K.K., Tokyo). Amplification was conducted in a total reaction volume of 20 μL containing 5 μL of cDNA, 10 μL of 2X SYBR Green PCR Master Mix, and .5 μL of each primer (10 μM). Primers for real-time reverse-transcription (RT) polymerase chain reaction (PCR) was used as shown in Table 1. Rabbit endogenous glyceraldehyde-3-phosphate dehydrogenase (GAPDH) gene expression was measured as an internal control and changes in each gene expression were expressed as the fold-increase relative to the control. All analyses were performed in triplicate. Amplifications were performed on a DNA Engine Opticon (MJ Research, Tokyo, Japan) using a DyNAmo^Tm^ SYBR^®^ Green qPCR kit (Finnzymes, MJ Bioworks, Inc., Espoo, Finland) according to the manufacturer’s instructions. The absence of non-specific amplification products was confirmed in a melting-curve analysis.

**TABLE 1:** Primer sequences and amplification conditions for real-time RT-PCR.

Gene	Primer sequence (5′-3′)	Annealing temperature (°C)	Product size (bp)
MMP-2	Gaa​ggt​caa​gtg​gtc​cgt​gt ccg​tac​ttg​cca​tcc​ttc​tc	68	167
MMP-3	Cgt​tcc​tga​tgt​tgg​tca​ctt​c ttg​gca​gat​ccg​gtg​tgt​aa	63	100
MMP-9	aaa​ctg​gat​gac​gat​gtc​tgc​gtc​ccg acc​tgt​tcc​gct​atg​gtt​aca​ccc​gcg​ta	58	362
MMP-12	tgg​cag​agg​tgg​tgt​cat​ag tgg​tca​cag​gca​gtt​ggt​tc	60	326
MCP-1	Ttcagctcccatgtgctt ctggacccacttctg	62	204
TIMP-1	gtc​atc​agg​gcc​aag​ttt​gt tcc​agc​gat​gag​aaa​ctc​ct	58	209
GAPDH	acg​gtg​cac​gcc​atc​act​gcc gcc​tgc​ttc​acc​acc​ttc​ttg	63	266

### 2.5 Western blot analysis

The abdominal aortic segments from 3 non-Tg and 3 Tg rabbits were homogenized in an ice-cold suspension buffer (10 mM Tris-HCl, pH7.6, 100 mM NaCl) supplemented with a proteinase inhibitor cocktail (Sigma, St. Louis, MO). The supernatant was collected, and the protein content was measured using a Bio-Rad protein assay kit (Bio-Rad Japan, Tokyo). Ten-microgram aliquots of the crude proteins from the artery were separated by 10% SDS-PAGE for Western blotting and probed with a panel of Abs against MMP-2, −3, −9, and −12 (Liang et al., 2006).

### 2.6 Statistical analyses

All values were expressed as the mean ± SE and statistical significance was analyzed using Student’s t*-*test or Mann-Whitney’s *U* test for non-parametric analysis. Statistical significance was set at *p* < .05.

## 3 Results

**FIGURE 2:**
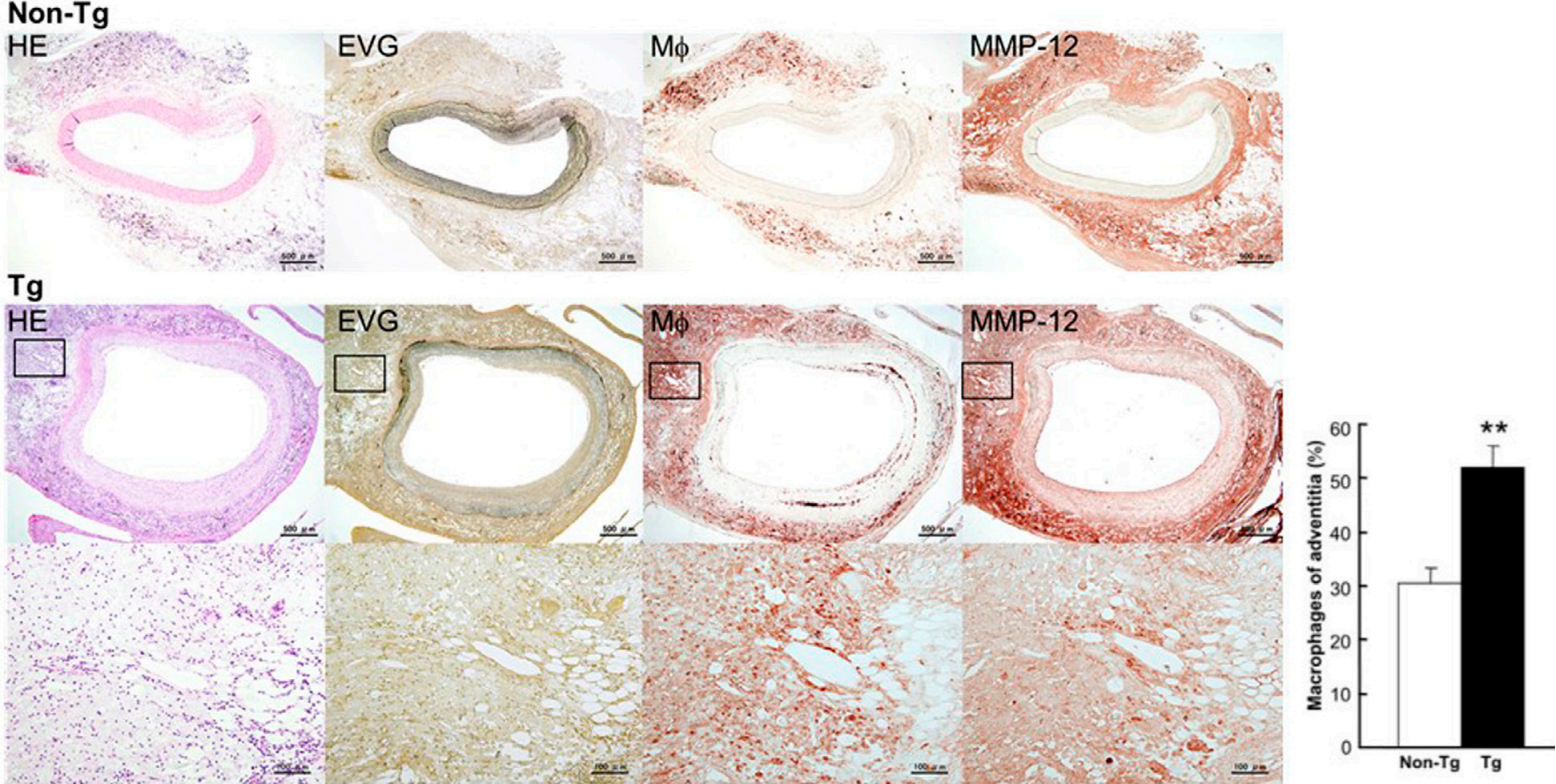
Micrographs of abdominal aortic lesions from non-Tg and Tg rabbits. Cross-sections were stained with H&E, with EVG, or immunohistochemically with Abs against macrophages and MMP-12. Adventitial macrophage infiltration and MMP-12 staining of a Tg rabbit aorta are shown at higher magnification at the bottom. Macrophage infiltration was quantitated and showed as percentage. 4–10 immunostained sections from each rabbit (*n* = 8 for non-Tg and 9 for Tg) were used. The data were expressed as the mean ± SE. **p < .01 vs. non-Tg.

By embedding carrageenan, we produced chronic inflammatory lesions around the abdominal aorta in rabbits fed a cholesterol diet. As shown in Figure 2, the peri-aortic lesions were mainly composed of infiltrating macrophages, many of which had a foamy appearance, resembling granulomatous lesions. We examined whether adventitial chronic inflammation influences aortic remodeling and intimal lesions or whether increased MMP-12 (derived from macrophages) is instrumental in these processes.

### 3.1 Adventitial lesions

**FIGURE 3:**
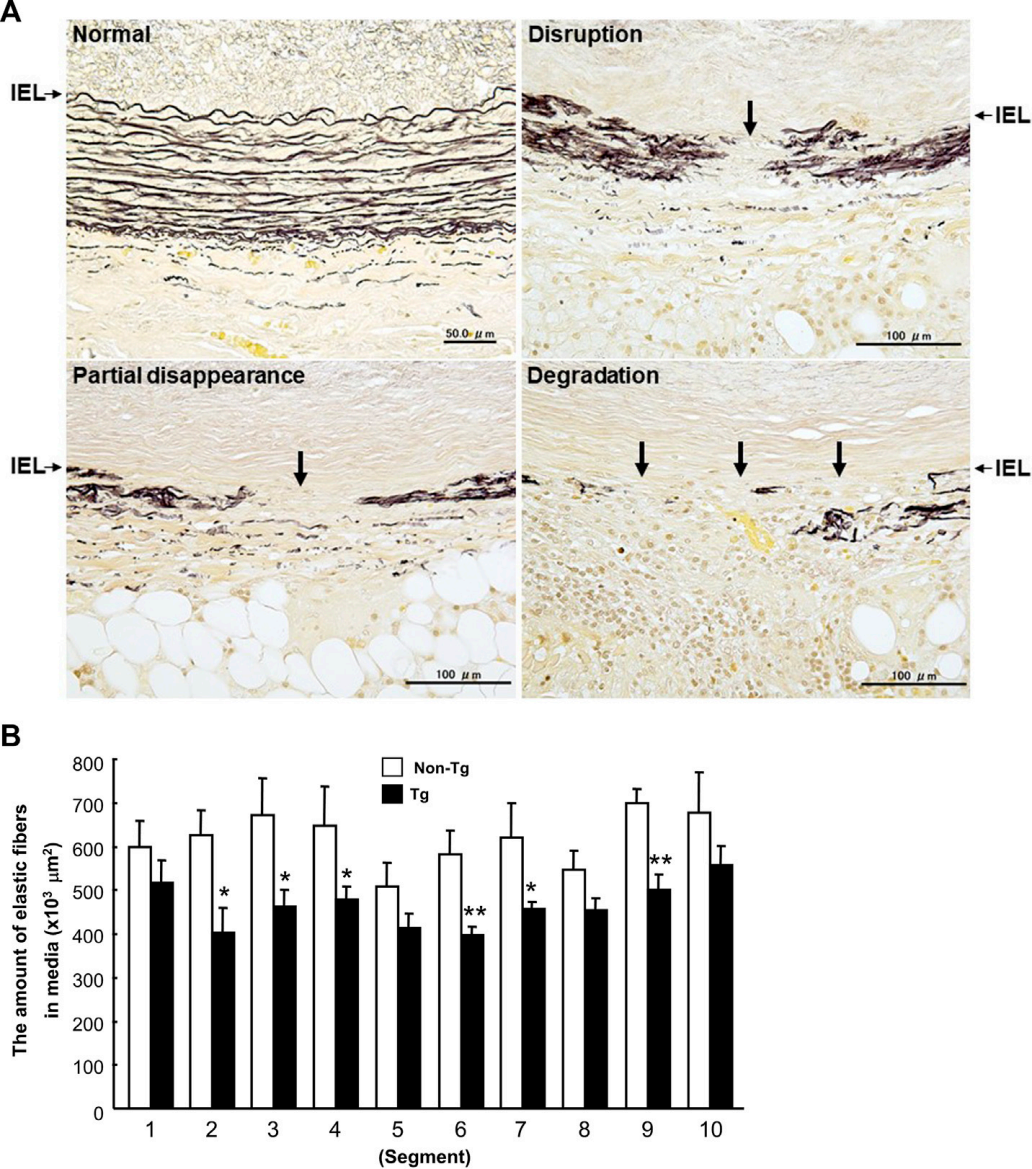
Disruption of elastin fibers of the aortic wall **(A)** and quantitation of the elastin contents of each section **(B)**. A: Histological features of destroyed elastic fibers in carrageenan-induced lesions. Cross-sections were stained with EVG. IEL (arrows): internal elastic lamina. B: The data were expressed as the mean ± SE (*n* = 12 for each group). *p < .05 **p < .01 vs. non-Tg.

**FIGURE 4:**
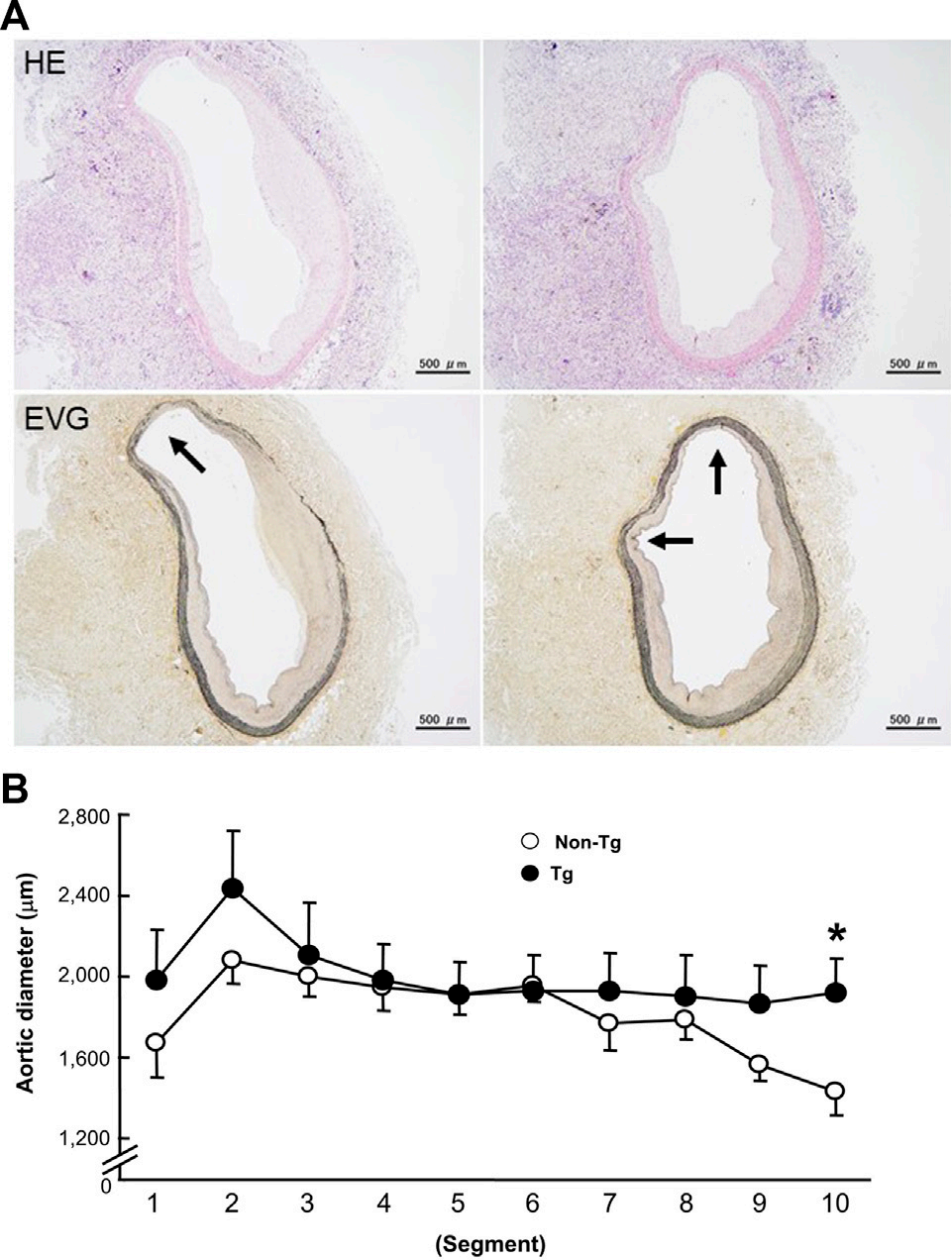
Representative micrographs of the focal dilation in the abdominal aortas of Tg rabbits **(A)** and quantitation of aortic dilation **(B)**. A: The lesions were stained with either H&E or EVG. Arrows indicate the protruded lumens. B: The aortic dilation was evaluated by measuring a defined area of the internal elastic lamina and calculating the diameter in each segment of the aorta. The data were expressed as the mean ± SE (*n* = 12 for each group). *p < .05 vs. non-Tg.

At first glance, the abdominal aortas were surrounded by numerous infiltrating macrophages with few intimal lesions (see below). The adventitial accumulation of macrophages was markedly and significantly increased in Tg compared to non-Tg rabbits and was associated with strong staining intensity of MMP-12 (Figure 2). *In situ* zymography confirmed that there was MMP-degrading activity around the aorta (data not shown). Furthermore, the lesions of Tg rabbits were characterized by remarkable disruption of the elastic fibers of both medial and adventitial lamellae as visualized with the EVG staining. The destruction of aortic elastin was histologically characterized by a partial or complete disappearance or degradation, or loss as shown in Figure 3A. In areas where the destruction occurred, there were many macrophages intermingled. To quantitate these changes, we measured the amount of elastin in each segment using EVG-stained specimens and compared Tg rabbits with non-Tg rabbits. The elastin content was significantly and consistently reduced throughout the segments of the abdominal aorta in Tg rabbits (Figure 3B). Of note, loss of the elastin was colocalized with MMP-12 immunoreactive proteins (Supplemental Fig.2) and associated with aortic remodeling in Tg rabbits. Under microscopic observation, we found that the aortic lumen focally protruded outwards and formed partially dilated (Figure 4A). We calculated the number of such lesions from all segments of the abdominal aorta and found that Tg rabbits had a 2-fold higher frequency of the dilated lesions than did non-Tg rabbits (4.0 ± .9 lesions/aorta in Tg vs. 2.2 ± .5 lesions/aorta in non-Tg, *p* < .05). We also measured the aortic diameter of each segment and found that 1–3rd and 7–10th of the segments of Tg rabbits were dilated compared to non-Tg aortas (Figure 4B) although average diameter of 10 segments were not statistically significant between two groups. Taken together, these results revealed that increased expression of MMP-12 in Tg rabbits resulted in more lesions and augmented degradation of the elastin in the aortic wall.

### 3.2 Intimal atherosclerosis

**FIGURE 5:**
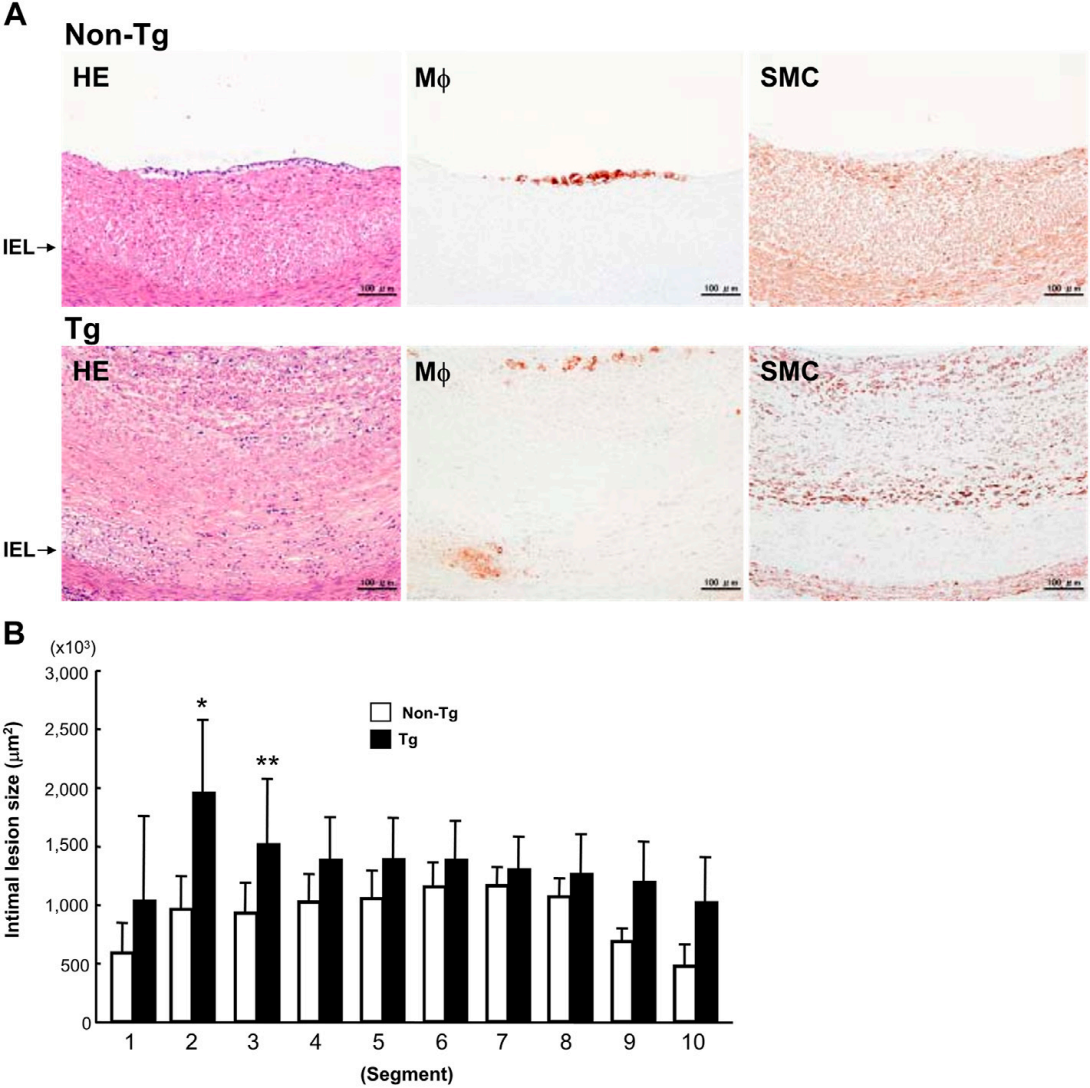
Representative intimal atherosclerotic lesions of Tg and non-Tg rabbits **(A)** and quantitation of intimal lesions **(B)**. A: Cross-sections of the aorta were stained with H&E or immunohistochemically stained with Abs against macrophages (Mϕ) and smooth muscle cells (SMC). B: The area occupied by intimal lesions was measured using an image analysis system as described in the Materials and Methods. The data were expressed as the mean ± SE (*n* = 12 for each group). *p < .05, **p < .01 vs. non-Tg.

To examine whether the adventitial inflammation also affects the abdominal aortic intima, we analyzed the intimal atherosclerotic lesions. The intimal lesions in the abdominal aortas were essentially characterized by intimal thickening and mainly composed of proliferating smooth muscle cells (SMCs) and extracellular matrix with very few macrophages at the surface (Figure 5A). We measured the area occupied by intimal lesions in each segment. As shown in Figure 5B, in all segments, the lesions tended to be larger in Tg than non-Tg rabbits.

### 3.3 Analysis of MMP expression

**FIGURE 6:**
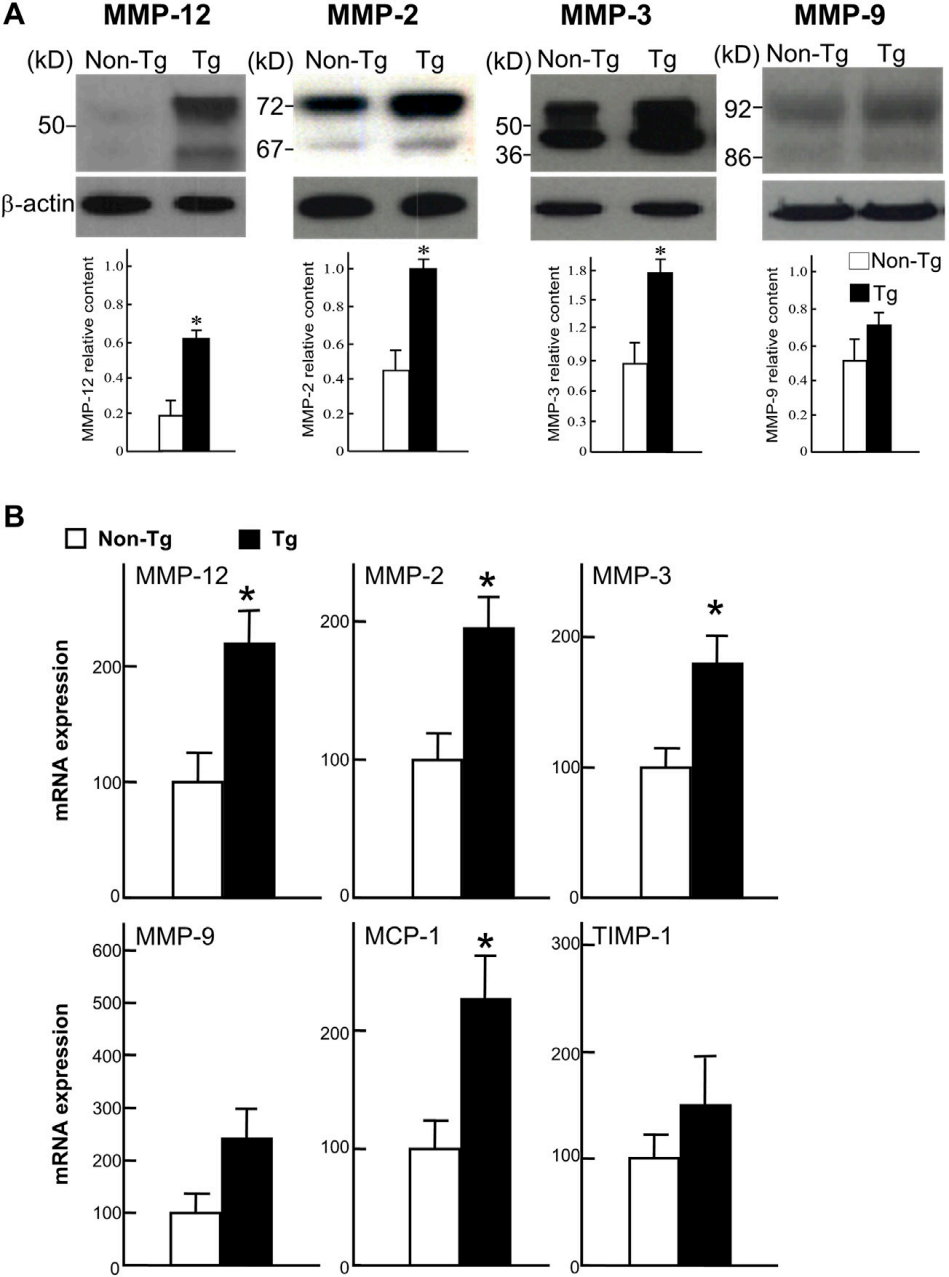
Western blot analysis **(A)** and real-time RT-PCR analysis **(B)**. A: Proteins isolated from the aortic lesions were subjected to 10% SDS-PAGE under reducing conditions, and probed with Abs against MMP-12, MMP-2, MMP-3, and MMP-9. The same membrane was re-probed with mAb against β-actin to indicate that equal amounts of proteins were loaded. The relative level of each MMP was quantitated by calculating the optical density (OD) of each signal on the films using a densitometer (GS-700, Bio-Rad) and normalized relative to the amount of protein for β-actin. All analyses were performed in triplicate and the values are expressed as the mean ± SE (*n* = 3 for each group). *p < .05 vs. non-Tg. B: Expression of MMP-12, -2, -3, and -9, MCP-1, and TIMP-1 were analyzed with real-time RT-PCR. Expression levels of each gene are expressed as a percentage of the control value. Data are expressed as the mean ± SE (*n* = 5 for each group). *p < .05 vs. non-Tg.

To investigate the status of MMPs in the lesions, we measured the MMPs expression at both the protein and mRNA levels using Western blotting and real-time RT-PCR. As shown in Figures 6A, B, levels of MMP-12 along with MMP-2 and -3 were significantly increased in Tg rabbits compared to non-Tg rabbits. The mRNA expression of monocyte chemoattractant protein-1 (MCP-1) was also increased but that of MMP-9 and TIMP-1 were not significantly changed in Tg rabbits.

## 4 Discussion

Although it is generally accepted that aortic atherosclerosis is the main risk factor for AAA (Alcorn et al., 1996; Golledge et al., 2006), the role of adventitial inflammatory macrophages, while involved in many vascular diseases (Michel et al., 2007), has not been fully appreciated. In this study, we developed a rabbit model of adventitial inflammation to investigate the influence of inflammatory macrophages and MMP-12 on abdominal aortic remodeling. We found that embedding carrageenan around the abdominal aorta for 24 weeks resulted in a marked accumulation of macrophages, many of which had a foamy appearance, histologically resembling granulomatous lesions. These macrophages could be possibly originated from the peritoneal cavity where many resident macrophages were normally present. It should be pointed out that the lesions produced by carrageenan differ from other models of AAA such as the periarterial application of calcium chloride in rabbit carotid arteries and aortas (Gertz et al., 1988; Freestone et al., 1997) because the carrageen treatment cannot lead to the formation of typical AAA lesions. However, this adventitial inflammation model allowed us to examine the potential role of inflammatory macrophages in the (1) destruction of the elastic lamellae of the aortic wall; (2) aortic remodeling (depicted as aortic dilation); (3) the formation of micro-aneurysm-like lesions; and (4) intimal thickening. In this respect, we were specifically interested in the role of MMP-12, an important elastase secreted from macrophages, which has been shown to play diverse roles in inflammatory processes such as arthritis (Wang et al., 2004), atherosclerosis (Johnson et al., 2005; Liang et al., 2006), and AAA (Curci et al., 1998; Longo et al., 2005).

Compared to non-Tg rabbits, Tg rabbits showed a greater infiltration of macrophages along with a greater reduction of the elastin content of the aortic wall, suggesting that MMP-12 derived from macrophages is indeed involved in the enhanced degradation of elastin. The decrease in elastin content of the aortic wall led to a partial dilation of aortic lumens. All these changes suggest that increased MMP-12 activity enhances aortic remodeling. Several possible mechanisms exist for these pathological changes in Tg rabbits. First, increased elastolytic activity of MMP-12 derived from adventitial macrophages may directly lead to the degradation of aortic elastin and other extracellular matrices. More importantly, in addition to MMP-12, other MMPs such as MMP-2 and -3 also derived from increased infiltrating macrophages may be upregulated in the lesions as shown by the results of Western blotting and real-time RT-PCR. Thus, it is likely that increased activity of MMP-12 may trigger the activation of other MMPs (Matsumoto et al., 1998) which work together in aortic remodeling. Second, increased MMP-12 may also result in increased macrophage infiltration through the generation of elastin peptide, which can induce monocyte chemotaxis (Senior et al., 1980). Of note, MCP-1, a potent chemoattractant for monocytes, was highly expressed in the lesions, which may indirectly indicate a high degree of inflammation. Lanone et al. (2002) demonstrated that MMP-12 can accelerate IL-13-induced inflammatory infiltration whereas MMP-12-deficient macrophages showed diminished proteolytic activity and migration (Shipley et al., 1996). Consistent with these observations, a deficiency in MMP-12 gene attenuated calcium chloride-induced aneurysm growth with less infiltration by macrophages in MMP-12 KO mice (Longo et al., 2005) although there was no effect on the elastase-infusion model (Pyo et al., 2000).

As mentioned above, we failed to observe gross aneurysms in the damaged abdominal aortas though we did observe dilated lesions under the microscope. This may be because in the current model, we covered the carrageenan-induced peri-aortic lesions with a sheet of polyethylene film to prevent inflammatory adhesion to the adjacent tissues, which may limit remarkable dilation of the aortic wall or aneurysms. Alternatively, the period of observation was not long enough for AAAs to form. Therefore, this model is merely an adventitial inflammatory model rather than a true AAA model. While having such a flaw, we could assess the influence of MMP-12 on aortic remodeling, which is closely associated with the formation of AAA. Recently, we also generated transgenic rabbits expressed high levels of MMP-1 and MMP-9 in macrophages (Niimi et al., 2019; Chen et al., 2020). It seems that MMP-1 is also involved in the formation of AAA but MMP-9 contributes the vascular calcification formation. In future, it needs to compare these MMPs in terms of AAA development.

In addition to the medial and adventitial lesions, we found that intimal atherosclerotic lesions were also more frequent in Tg rabbits than non-Tg rabbits. However, these intimal lesions were essentially characterized by the proliferation of SMCs whereas macrophage infiltration was less prominent. This result was surprising because the SMC-rich lesions induced by adventitial inflammation were sharply different from the foam cell-rich lesions often observed in the aortic arch and thoracic aortas of cholesterol-fed rabbits (Liang et al., 2006). It seems that the SMC-rich lesions seen in these rabbits are more like injury-induced (balloon or cuff) lesions (Booth et al., 1989; Kockx et al., 1993). It has been reported that enhanced elastin fragmentation is required for the migration and proliferation of SMCs (Zempo et al., 1994; Southgate et al., 1996). Because we merely examined the aortic lesions at 24 weeks after surgery, it is also possible that macrophage-rich lesions are present in the early stages.

In conclusion, we have demonstrated that adventitial inflammation can affect aortic remodeling, and increased activity of MMP-12 derived from infiltrating macrophages leads to the enhanced formation of lesions. These results suggest that adventitial inflammation is a crucial stimulus for the development of vascular lesions and remodeling. Our findings clearly support the hypothesis that MMP-12 derived from adventitial macrophages plays an important role in the pathogenesis of atherosclerotic disease such as AAA. It would be interesting to examine whether the administration of a MMP-12 specific inhibitor (Devel et al., 2006) prevents the formation of atherosclerosis *in vivo*.

## Data Availability

The raw data supporting the conclusion of this article will be made available by the authors, without undue reservation.
