# Supplementary material for: Macrophage elastase derived from adventitial macrophages modulates aortic remodeling
Source: Front Cell Dev Biol. 2023 Jan 10;10:1097137. doi: 10.3389/fcell.2022.1097137 (PMC9871815; doi:10.3389/fcell.2022.1097137)
Supplement: Supplementary file 1 [file Presentation1.PPT]

## Slide 1
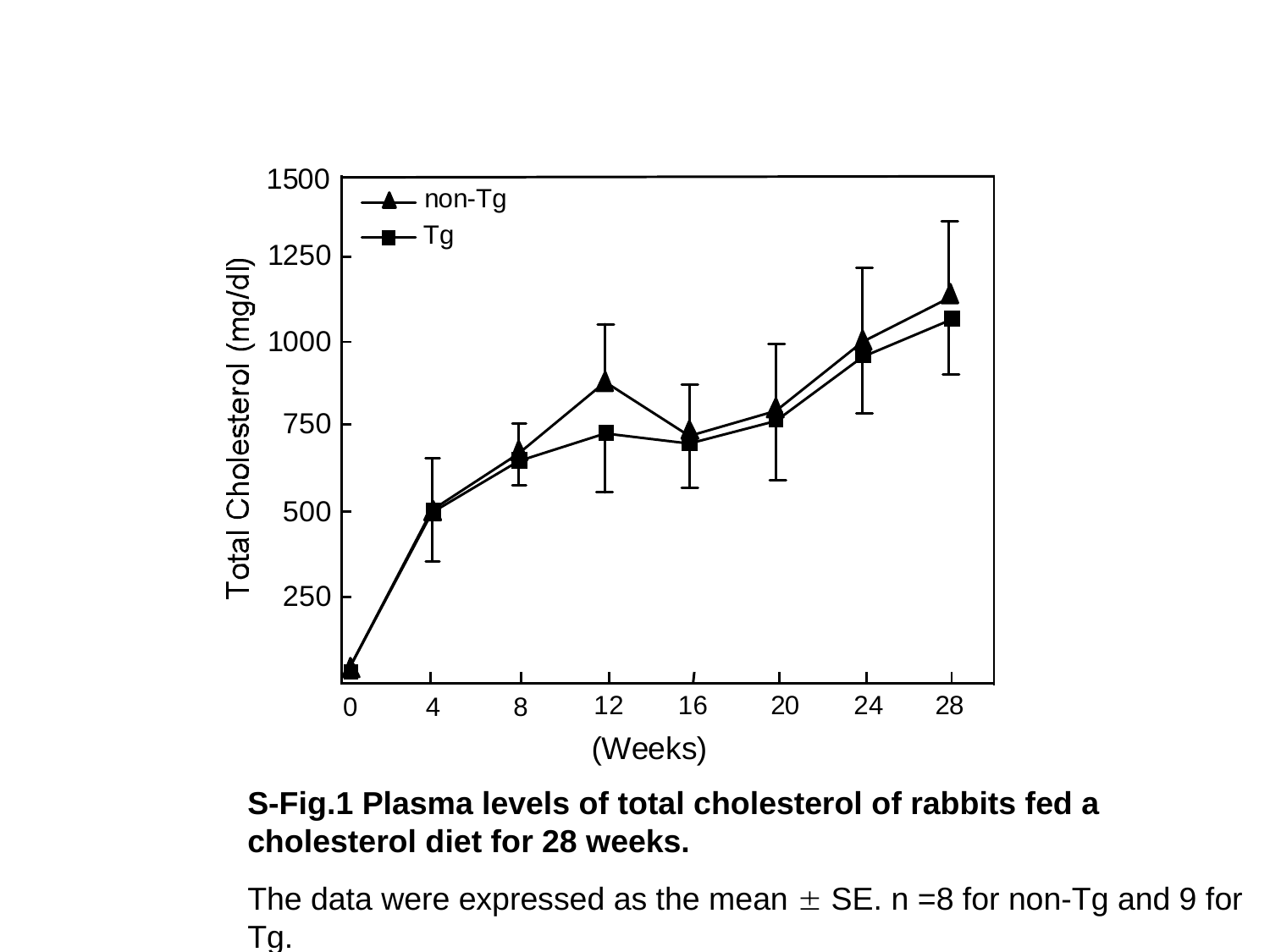

S-Fig.1 Plasma levels of total cholesterol of rabbits fed a cholesterol diet for 28 weeks.
The data were expressed as the mean ± SE. n =8 for non-Tg and 9 for Tg.
