# Supplementary material for: Macrophage elastase derived from adventitial macrophages modulates aortic remodeling
Source: Front Cell Dev Biol. 2023 Jan 10;10:1097137. doi: 10.3389/fcell.2022.1097137 (PMC9871815; doi:10.3389/fcell.2022.1097137)
Supplement: Supplementary file 2 [file Presentation2.PPTX]

## Slide 1
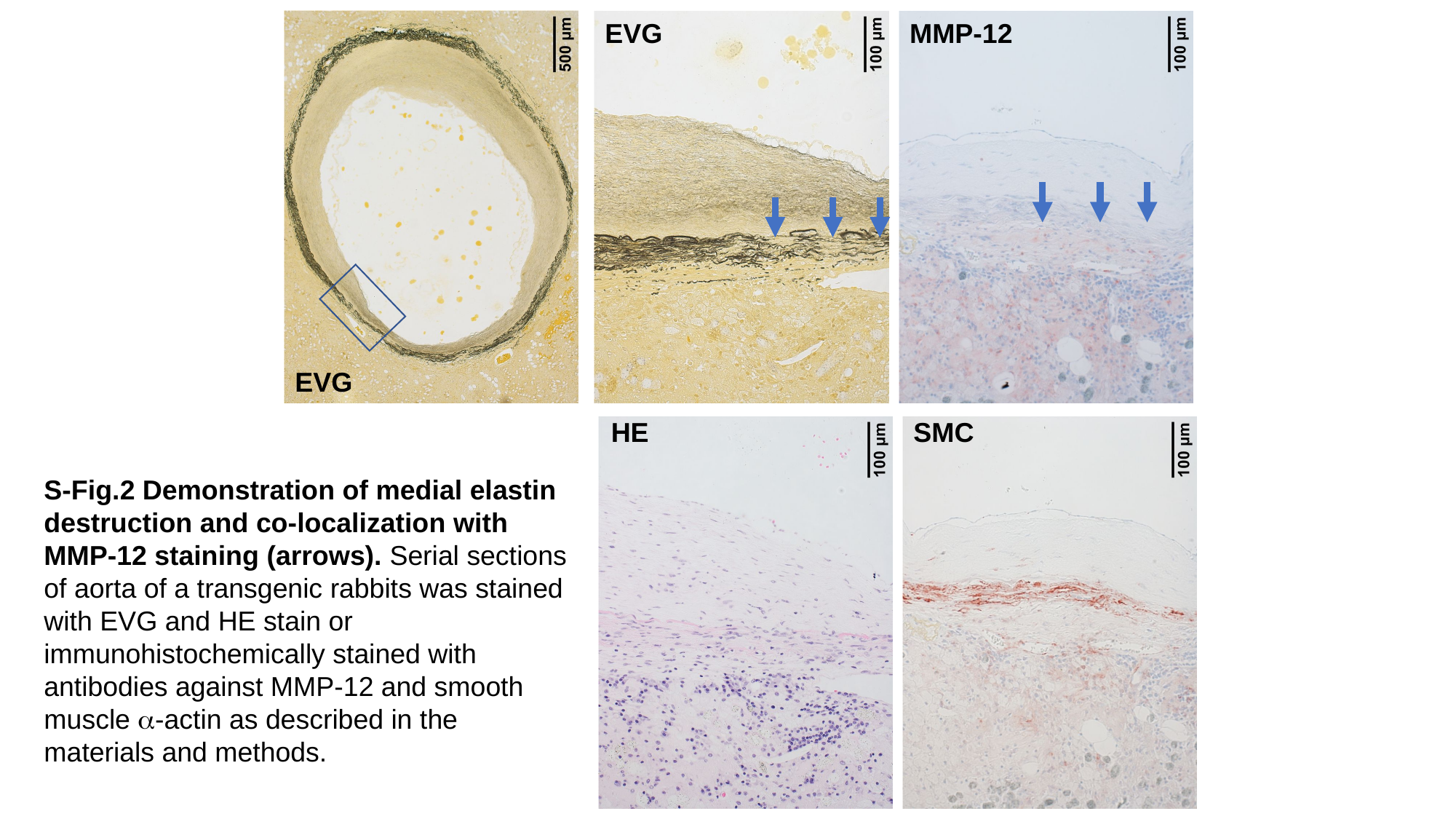

EVG
MMP-12
EVG
HE
SMC
S-Fig.2 Demonstration of medial elastin destruction and co-localization with MMP-12 staining (arrows). Serial sections of aorta of a transgenic rabbits was stained with EVG and HE stain or immunohistochemically stained with antibodies against MMP-12 and smooth muscle a-actin as described in the materials and methods.
